# Identification of gene signatures related to hypoxia and angiogenesis in pancreatic cancer to aid immunotherapy and prognosis

**DOI:** 10.3389/fonc.2023.1119763

**Published:** 2023-03-30

**Authors:** Xiushen Li, Xi Yang, Weiqi Xue, Rui Yang, Zhiwei He, Lisha Ai, Hui Liu

**Affiliations:** ^1^ Department of Obstetrics and Gynaecology, Shenzhen University General Hospital, Shenzhen, Guangdong, China; ^2^ Guangdong Key Laboratory for Biomedical Measurements and Ultrasound Imaging, National-Regional Key Technology Engineering Laboratory for Medical Ultrasound, School of Biomedical Engineering, Shenzhen University Medical School, Shenzhen, Guangdong, China; ^3^ Shenzhen Key Laboratory, Shenzhen University General Hospital, Shenzhen, Guangdong, China; ^4^ Department of Ultrasound, The People’s Hospital of Shapingba District, Chongqing, China; ^5^ The First Clinical Medical College, Guangzhou University of Chinese Medicine, Guangzhou, Guangdong, China; ^6^ Department of Hepatobiliary Surgery, Shenzhen University General Hospital, Shenzhen University, Shenzhen, Guangdong, China; ^7^ Department of Teaching and Research, Shenzhen University General Hospital, Shenzhen University, Shenzhen, Guangdong, China; ^8^ Guangdong Provincial Key Laboratory of Regional Immunity and Diseases and Carson International Cancer, Shenzhen University, Shenzhen, China

**Keywords:** pancreatic cancer, immunotherapy, hypoxia, angiogenesis, prognostic model

## Abstract

**Background:**

One of the most diverse tumors is pancreatic cancer (PC), which makes predicting the prognosis challenging. PC development is directly related to hypoxia, angiogenesis, and immunotherapy. It is still unclear how the three features are related.

**Methods:**

The Genotype-Tissue Expression (GTEx) and the Cancer Genome Atlas (TCGA) database were employed to obtain sequencing data for healthy pancreatic tissues and PC tissues, respectively. According to the constructed hypoxic prognostic model (HPM) and angiogenic prognostic model (APM), 4 subtypes of PC were identified. Hypoxia and angiogenesis prognostic model (HAPM) was established based on differentially expressed genes (DEGs) between high-angiogenesis/high-hypoxia (HH) and low-angiogenesis/low-hypoxia (LL) subgroups. Base on the median risk score, PC patients were separated into high-risk and low-risk groups, and clinical traits, prognosis, percentage of immune cell infiltration, PD-1 expression, and the fraction of T-cell depletion were compared between the groups. Finally, the predictive accuracy of the tumor immune dysfunction and rejection (TIDE) and tumor inflammatory signature (TIS) models, as well as HAPM, was compared.

**Result:**

We analyzed the mRNA sequencing data from 178 PC tissues and 171 normal pancreatic tissues to obtain 9527 DEGs. We discovered 200 genes linked with hypoxia and 36 genes involved with angiogenesis through the literature. We found the core genes related with hypoxia and angiogenesis in PC by intersecting the DEGs of the HH and LL subgroups with those of PC *via* WGCNA. IL-17 signaling pathway, ECM-receptor interactions, cytokine receptor interactions, etc. were all enriched in the Kyoto Encyclopedia of Genes and Genomes (KEGG) results of core genes. HAPM has good predictive efficiency, according to an evaluation of KM survival curves and ROC curves. The external dataset also validated the model’s ability to anticipate outcomes. Patients in the high- and low-risk groups were compared for PD1 expression and T-cell exclusion scores, which suggested that the model might be used to forecast which PC patients might benefit from immunotherapy.

**Conclusions:**

The probable molecular processes connecting hypoxia and angiogenesis are described in this work, and a model is developed that may be utilized to forecast the prognosis for PC patients and the benefits of immunotherapy.

## Introduction

Pancreatic cancer (PC) has an insidious onset, rapid progression, extremely poor treatment outcome, and a parallel increasing trend of morbidity (1). PC is currently thought to be a multifactorial disease, which refers to a complex interaction between cancer cells, the microenvironment, and the person, including genetic, metabolic, immunological, etc (2, 3). As a result, the popular research area now includes the investigation of PC etiology, early detection, medication screening, treatment plan, and prediction of recurrence and metastasis.

One of the traits of cancer is angiogenesis, a process that facilitates rapid tumor development, allows for remote tumor spread, and feeds tumor tissue with the oxygen and nutrients needed for metabolism (4). Angiogenesis is one of the major contributors in tumor growth, infiltration and metastasis (5). Neovascularization, which develops inside tumor tissues and feeds tumor cells with nutrients, causes a rapid rise in the number of tumor cells, their volume, their infiltration of nearby tissues, and their propensity to metastasize (6). Since the first hypothesis that tumor growth is dependent on angiogenesis, the study of angiogenesis in solid tumors has drawn an increasing number of academics, and inhibiting tumor angiogenesis has emerged as a key method for modern tumor treatment (7). Tumors need to rely on their surrounding blood vessels to maintain nutrition, and once the vascular supply is blocked, the tumor will be killed (8). Pro-angiogenic and anti-angiogenic factors regulate the incredibly complex process of tumor angiogenesis (9). An angiogenic switch is created by the dynamic balance of these elements, and it closes when the activity of pro- and anti-angiogenic factors is balanced (10).

The most crucial metabolic characteristic that sets malignant tumors from normal tissues is that tumor cells are frequently starved due to their energy depletion, which far outweighs the energy supply they can acquire (11). Thus, as tumors grow, nutrient depletion and hypoxia within the tumor tissue are common features of most tumors (12). Since of chaotic vascular architecture, areas of necrosis, lack of oxygen diffusion from developing, enlarging tumors, and uneven blood flow, hypoxia may be present throughout the tissue in solid tumors (11). Hypoxia is a crucial element of the solid tumor microenvironment (TME) and is linked to a number of characteristics of cancer, such as metabolic reprogramming, impaired immune response, increased genomic instability, tumor malignant progression, treatment resistance, and poor clinical outcomes for patients (13). In the hypoxic microenvironment of pancreatic cancer, HIF-1A is a key transcription factor during hypoxia and can often transcriptionally upregulate the expression of angiogenesis-related genes such as VEGFR and FGFR. However, the infiltration of immune cells during tumor microangiogenesis also contributes to the immunosuppressive microenvironment of pancreatic cancer (14, 15). When the normoxia-hypoxia transition occurs, cells predominantly rely on HIF signaling, and they adjust to the hypoxic environment by turning on HIF signaling (16). Hypoxia is often accompanied by disruption of cancer-related features, such as reprogramming of intracellular transcriptional programs, up- or down-regulation of the expression of certain genes and proteins, which allows cells to escape apoptosis and promotes cell migration to more oxygenated areas (17). The hypoxic microenvironment induces genomic instability and altered DNA repair, such as increased mutational load, often seen in hypoxic tumors (18). In order to induce angiogenesis, extracellular matrix remodeling, and growth factor signaling, tumor hypoxia encourages the recruitment of endothelial, pericytes, and stromal cells (19). Different cell types react to hypoxia in various ways, and when combined, they support a tumor development environment that is immune tolerant. Since hypoxia evades immune monitoring through a variety of mechanisms, therapeutic methods to stimulate anti-tumor immunity are ineffective (20). Indeed, the advancement of immunological research has shed light on the tumor microenvironment as a promising factor in reactivating the immune response against cancer cells, causing a paradigm shift in the field of oncology. Nevertheless, the intricacy of the tumor microenvironment implies the involvement of multiple pathways connecting to immunomodulatory mechanisms (21). Acidification of the extracellular environment mediated by hypoxia impedes T-cell expansion or inhibits T-cell toxic effector functions, resulting in low or absent numbers of effector T cells in TME and poorer T-cell immune checkpoint receptor PD-1 blockade effects in some tumors (22). In summary, hypoxia plays an essential function in establishing and maintaining tumor immune effects or immunotherapy.

Although the regulatory mechanisms are still unclear, recent investigations have demonstrated the complicated relationship between the hypoxic microenvironment, neovascularization, and immune modulation in PC tissue. In this study, the PC hypoxia and neovascularization dataset that was made available in public databases was used to build the hypoxia and angiogenesis prognostic model (HAPM), and the relationship between this model and immunotherapy was further examined. The novel theoretical foundation for PC immunotherapy is provided by the hypoxia and angiogenesis prognostic model, which also analyzes the regulatory targets and downstream signaling cascades.

## Materials and methods

### Data download and processing

Sequencing data from healthy and cancerous pancreatic tissue were obtained using the databases Genotype-Tissue Expression (GTEx) and The Cancer Genome Atlas (TCGA). The R “limma” and “ggplot2” packages were used to select and display differentially expressed genes (DEG). The GEO database’s GSE62452 and GSE78229 datasets were downloaded in order to validate the model’s predictive capabilities later on.

### Establishment of HPM and APM

The hypoxic prognostic model (HPM) was constructed as follows (1): review the literature through pubmed website to obtain hypoxia-related genes (2); take the intersection of hypoxia-related genes and PC DEG (3); sequentially construct HPM by univariate Cox, last absolute shrinkage and selection operator (LASSO), multivariate Cox analysis was performed to construct HPM (4); calculate the patient’s risk score calculated according to the prognostic model (5); divide patients into high and low risk groups based on the median value of the risk score (6); assess the prognostic efficacy of the prognostic model though Kaplan-Meier (KM) survival curve and receiver operating characteristic (ROC) curve. The angiogenic prognostic model (APM) was created in a way that was consistent with HPM.

### Clinical and immunological characteristics of various subgroups of patients

Based on the patients’ angiogenesis and hypoxia risk scores, four subgroups were created: high-hypoxia/high-angiogenesis (HH), high-hypoxia/low-angiogenesis (HL), low-hypoxia/high-angiogenesis (LH) and low-hypoxia/low-angiogenesis (LL). The KM survival curve was applied to examine the variations in prognosis among the various patient categories. Heat maps were employed to show the variations between the clinical traits of the various patient subgroups. The “CIBERSORT” function and the “gsva” function were utilized to calculate immune cell infiltration and immune-related functions for various patient subgroups, and the results were displayed as box plots.

### Construction of HAPM

DEGs from PC and DEGs between the HH and LL subgroups were intersected. The intersecting gene modules with the greatest significance to PC were screened by using weighted gene co-expression network analysis (WGCNA) algorithm. We examined genes contained in gene modules using PPI networks, GO, and KEGG enrichment analyses in order to uncover crucial targets and biological processes involved with hypoxia and angiogenesis in PC. HAPM was then created using the same techniques used to develop hypoxia prognostic models.

### Comparison of the predictive efficacy of HAPM with clinical traits

We sequentially conducted univariate Cox and multivariate Cox analyses using the clinical characteristics of PC patients to determine whether HAPM may be a predictive factor irrespective of the patient’s clinical qualities. ROC curves and C-indices were used to compare the prediction efficacy of clinical traits and HAPM. Heat maps depict the distribution of clinical features among various patient subgroups.

### Comparison of immune-related gene expression in different HAPM subgroups

Box plots are used to demonstrate the expression of immune-related genes in different subgroups of patients. Immune-related genes mainly contained genes related to Major Histocompatibility Complex, Inhibitory Immune Makers, Activation Immune Makers, Anti-inflammatory Cytokines Makers, and pH Regulation Maker.

### Relevance of HAPM to immunotherapy

Compare the distribution of immune types between different risk groups and visualize using heat maps and sankey plots. Visualize PD-1 expression and T-cell exclusion scores for patients in different risk subgroups using box plots and calculate the correlation between PD-1 expression and risk scores. To evaluate the prognostic efficacy of HAPM with that of the TIDE and TIS models, ROC curves were used.

## Results

### Scanning the PC for DEG

A total of 9527 DEGs were found using the screening criteria (|logFC|>0.585, fdr value 0.05). Gene expression was visualized *via* volcano plots, with black nodes designating genes with no discernible change in expression and blue and red nodes designating genes with low and high expression in tumor samples, respectively (**Supplementary Figure 1A**). The 50 most differentially expressed up- and down-regulated genes in each sample were then shown in the form of a heat map (**Supplementary Figure 1B**).

### Construction and validation of HPM

**Figure 1:**
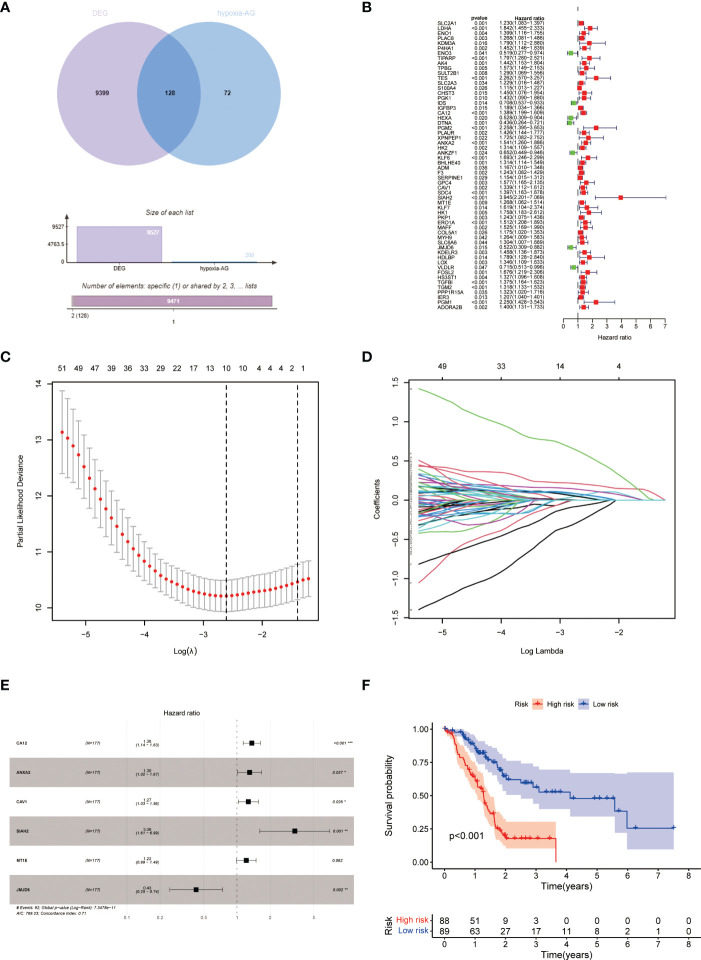
Construction of HPM. **(A)** Venn diagram of hypoxia-associated genes and DEG of PC; **(B–E)** Construction of h HPM using univariate Cox, lasso, and multivariate Cox analyses. **(F)** KM survival curves of HPM.

After reviewing literature, we obtained a total of 200 hypoxia-associated genes (**Supplementary Table 1**). 128 hypoxia-related DEGs were created by intersecting the hypoxia-related genes with DEGs (**Figure 1A**). 58 genes were then identified using the univariate Cox analysis (**Figure 1B**). Then, using LASSO analysis, 10 genes were discovered after 1000 cross-validations (**Figures 1C, D**). After doing multivariable Cox analysis, we were able to create the hypoxic predictive model using 6 genes (**Figure 1E**), which included 5 risk factors (CA12, ANXA2, CAV1, SIAH2, and MT1E) and 1 protective factor (JMJD6). We created KM survival curves and ROC curves to evaluate the prognostic effectiveness of the model, and findings revealed that HPM had a strong prognostic efficacy (**Figure 1F** and **Supplementary Figure 2G**). Survival analysis of the 6 genes used to construct the model showed that CA12, ANXA2, CAV1, SIAH2, and MT1E were associated with poor prognosis (**Supplementary Figures 2A–E**), whereas JMJD6 was associated with favorable prognosis (**Supplementary Figure 1F**), in line with the model.

### Construction and validation of APM

**Figure 2:**
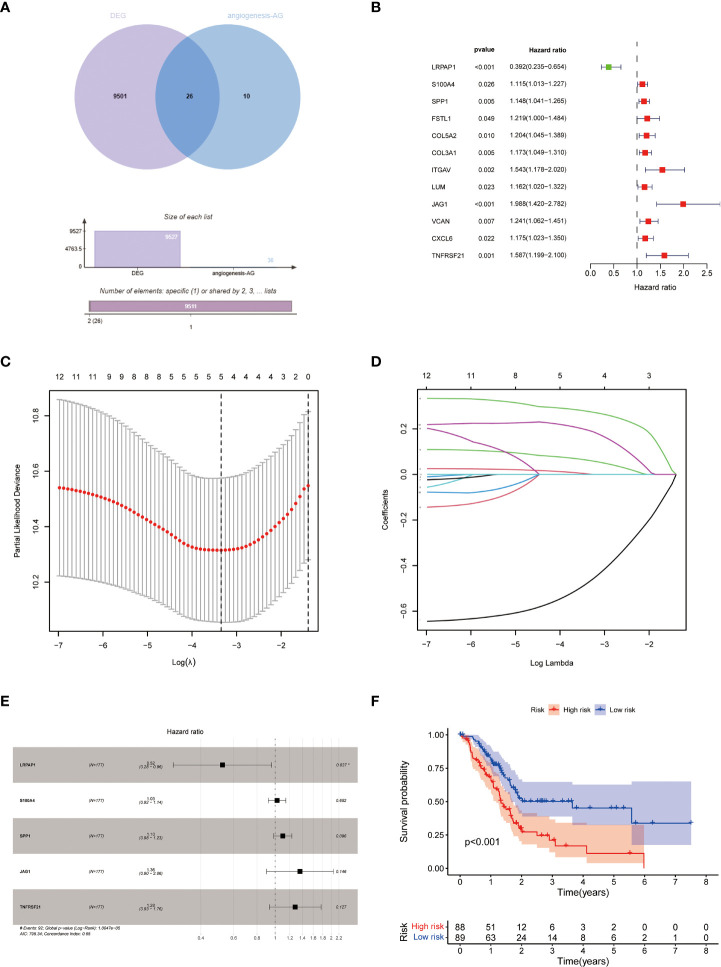
Construction of APM. **(A)** Venn diagram of angiogenesis-related genes and PC DEG; **(B–E)** Construction of APM using univariate Cox, lasso, and multivariate Cox analyses. **(F)** KM survival curves of APM.

APM was constructed and validated in line with HPM. A total of 36 angiogenesis-related genes were obtained (**Supplementary Table 2**). After taking intersection with DEG, 28 DEG associated to angiogenesis were discovered (**Figure 2A**). We eventually arrived at APM consisting of 5 genes (**Figures 2B–E**), with S100A4, SPP1, JAG1, and TNFRSF21 as risk factors and LRPAP1 as a protective factor, by progressively performing univariate Cox, LASSO, and multivariable Cox analyses (**Supplementary Figures 3A–E**). The prognostic efficiency of APM was demonstrated by KM survival curves and ROC curves (**Figure 2F** and **Supplementary Figure 3F**). According to the model, lRPAP1 was linked to a positive prognosis (**Supplementary Figure 3A**), but S100A4, SPP1, JAG1, and TNFRSF21 were linked to a negative prognosis (**Supplementary Figures 3B–E**), consistent with the model.

### Results of the differential analysis of clinical and immunological characteristics of the 4 subgroups

**Figure 3:**
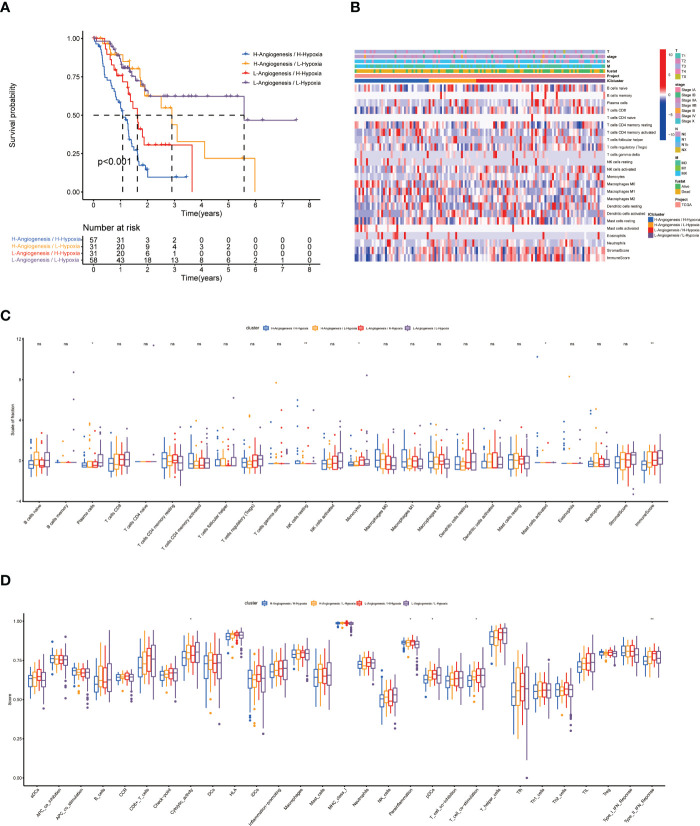
Prognosis and related immunological information for patients with 4 subtypes. **(A)** KM survival curves for 4 subtypes. **(B)** Heat map of the distribution of clinical information and immune cell ratios in 4 subtypes. **(C, D)** Differential analysis of immune cell ratios and immune cell function in 4 subtypes. * indicates a significant difference between groups, p<0.05; ** indicates a significant difference between groups, p<0.01; "NS" no significant difference between groups, p≥0.05.

The results of KM survival curves revealed that HH subgroup had a significantly better prognosis than LL subgroup (**Supplementary Table 3**; **Figure 3A**). We used heatmap to demonstrate the distribution of clinical characteristics and immune cell ratios in the 4 subgroups (**Figure 3B**). To compare the differences in immune cell proportions and functions between subgroups, we visualized them by using box-line plots. The results revealed that the proportions of Plasma cells, NK cells resting, Monocytes, Mast cells activated and the functions of Cytolytic activity, Para inflammation, pDCs, T cell co stimulation, and Type II IFN Response were significantly different (**Figure 3C**, **D**).

### Results of screening and bioinformatics analysis of core hypoxia and angiogenesis genes

**Figure 4:**
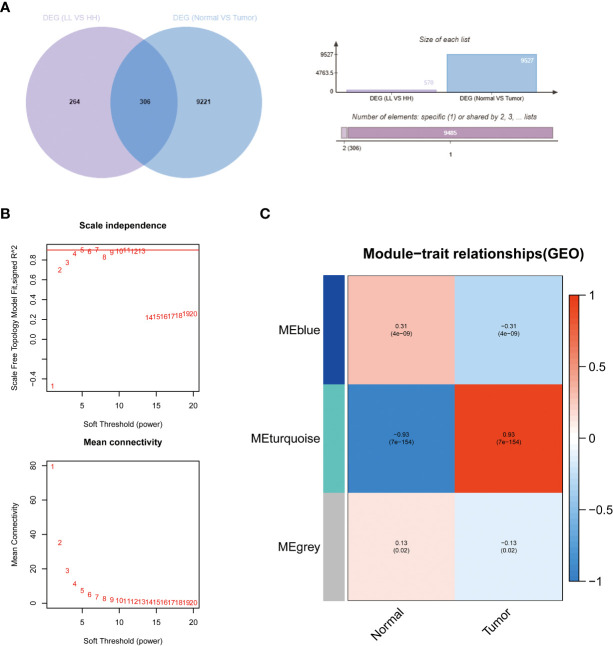
The results of WGCNA. **(A)** Venn diagrams of DEG (LL vs HH) and PC DEG. **(B)** Confirmation of the soft threshold for WGCNA. **(C)** Correlation analysis of relevant gene modules with PC.

We screened 570 DEGs containing 140 down-regulated genes and 430 down-regulated genes from the HH subgroup and LL subgroup. To screen out the core genes associated with hypoxia and angiogenesis in PC, we intersected these DEGs with those of PC (**Figure 4A**). The 306 intersecting genes we retrieved were subjected to WGCNA (**Figure 4B**). As shown in **Figure 4C**, the MEturquoise gene module had the highest correlation with PC, so we considered this fraction of genes as the core genes associated with hypoxia and angiogenesis in PC.

**Figure 5:**
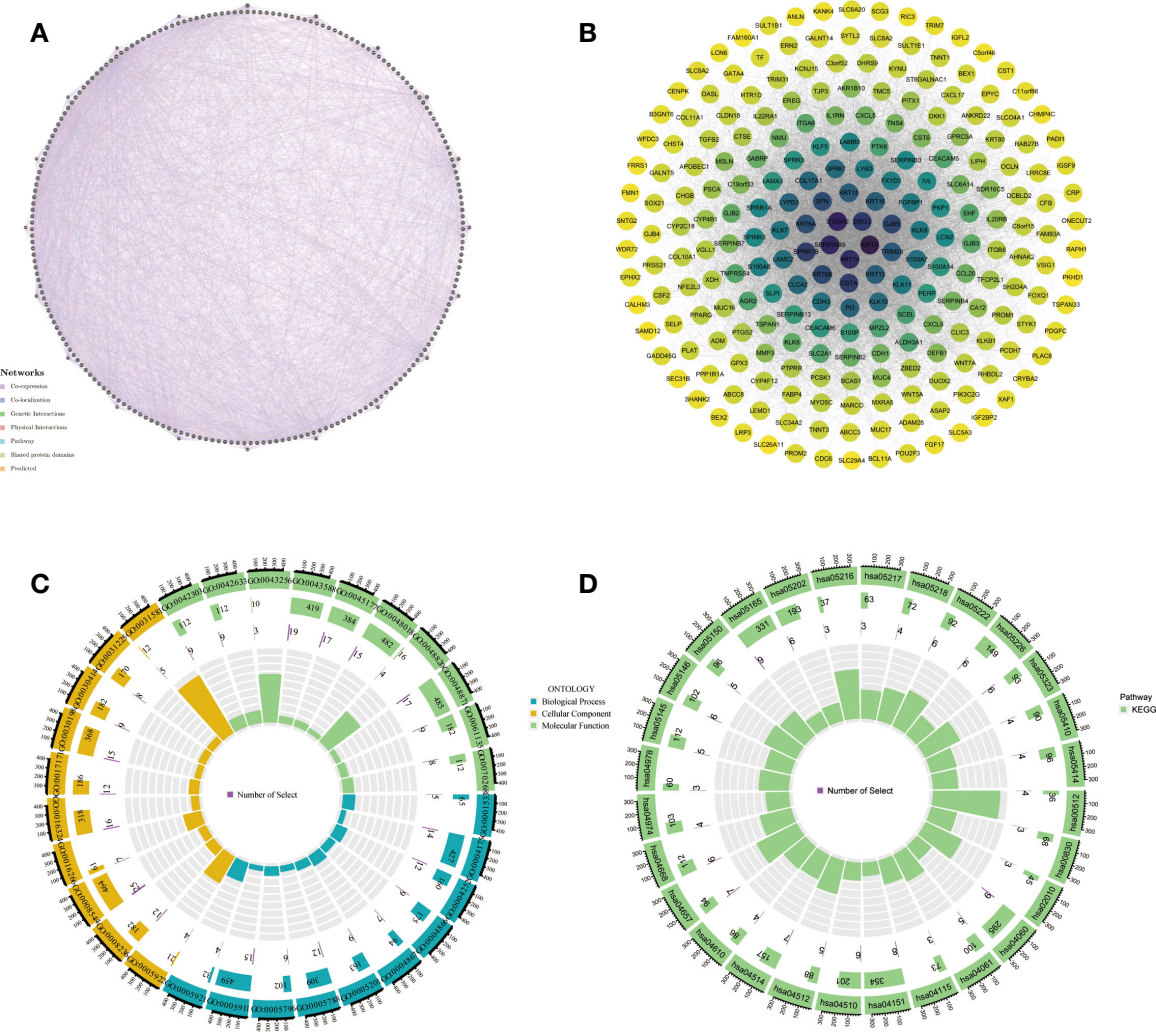
Bioinformatics analysis of genes obtained by WGCNA. **(A, B)** PPI network, **(C, D)** GO and KEGG enrichment analysis.

Genes from the MEturquoise module were uploaded into the GeneMANIA database to construct the PPI network (**Figure 5A**; **Supplementary Table 5**). To screen out the core genes, we imported the analysis results into Cytoscape software for analysis and found that S100A2, DSG3, KRT5, KRT14, SERPINB5 may be significantly associated with hypoxia and angiogenesis (**Figure 5B**). Following that, we then performed Gene Ontology and Kyoto Encyclopedia of Genes and Genomes (KEGG) enrichment analysis of genes in the MEturquoise module (**Figures 5C, D**; **Supplementary Tables 6**, **7**). Results of molecular function enrichment analysis were enriched in epidermal development, hemidesmosome assembly, skin development, molting cycle, hair cycle, O-glycan processing, extracellular matrix organization, hair follicle maturation, multicellular organism homeostasis, cornification, etc. Results of KEGG enrichment analysis were enriched for IL-17 signaling pathway, mucin-type O-glycan biosynthesis, amoebiasis, TNF signaling pathway, ECM-receptor interactions, Staphylococcus aureus infection, viral proteins interacting with cytokines and cytokine receptors, etc.

### Construction and validation results of HAPM

**Figure 6:**
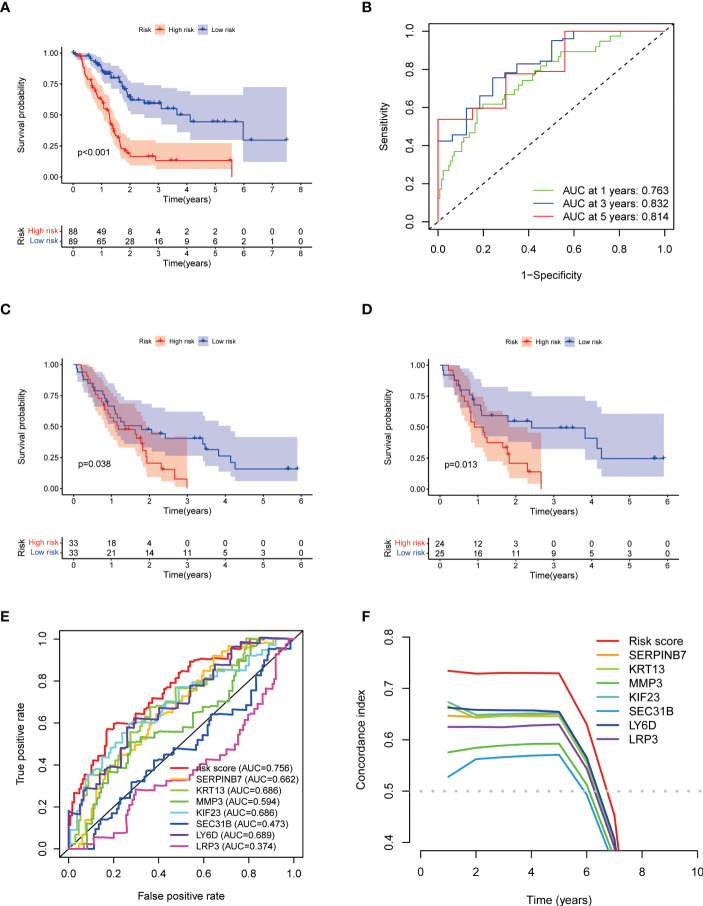
Construction and validation of HAPM. **(A, B)** KM-survival curve and ROC curve for the TCGA-PAAD dataset. **(C, D)** KM-survival curves of the GSE62452 and GSE78229 dataset (validation). **(E, F)** ROC curves and C-indices for HAPM and genes constructing HAPM (TCGA-PAAD).

HAPM was constructed and validated in the same way as HPM (**Supplementary Figures 4A–D**; **Supplementary Table 4**), resulting in the construction of HAPM consisting of 7 genes. The results of the KM survival curves and ROC curves showed that HAPM could be used to predict the prognosis of PC patients and had good predictive power (**Figures 6A, B**). The validation results of the GSE62452 and GSE78229 datasets likewise demonstrated HAPM’s strong predictive power (**Figures 6C, D**). the comparison of the predictive performance of HAPM with the constructed model genes also showed strong predictive power (**Figures 6E, F**).

### Results of comparison of HAPM with clinical traits

**Figure 7:**
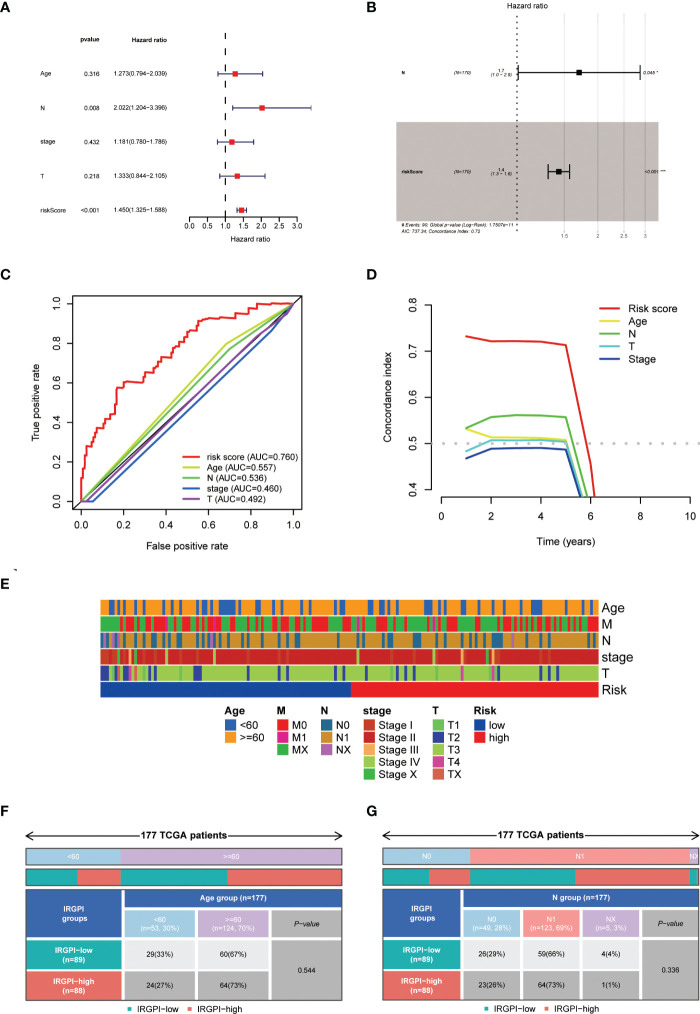
Relationship between HAPM and clinical characteristics. **(A, B)** univariate Cox and multivariate Cox analyses of the risk score and clinical characteristics. **(C, D)** Comparison of prognostic efficacy of the risk score with clinical characteristics by ROC curves and C-indices. **(E)** Distribution of clinical characteristics across different subgroups. **(F, G)** Differences in age and N-stage in different subgroups.

We found that risk score was able to act as a prognostic factor independent of the patient’s clinical traits (**Figures 7A, B**). We found that the prognostic efficacy of the model was significantly better than that of the patient’s clinical traits (**Figures 7C, D**). No discernible relationship between risk scores and clinical qualities was discovered by examining the distribution of clinical traits among different subgroups (**Figures 7E–G**; **Supplementary Figures 5A, B**).

### Results of correlation analysis of HAPM with immunotherapy

**Figure 8:**
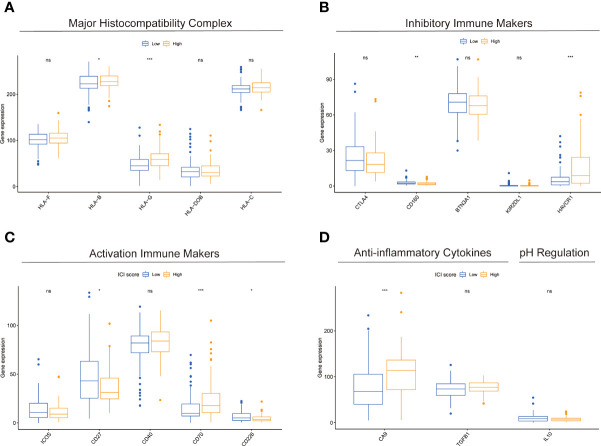
Comparison of immune-related markers between subgroups based on HAPM. **(A–D)** The differences in the expression of genes related to Major Histocompatibility Complex, Inhibitory Immune Makers, Activation Immune Makers, Anti-inflammatory Cytokines, and pH Regulation in different subgroups of patients, respectively.

**Figure 9:**
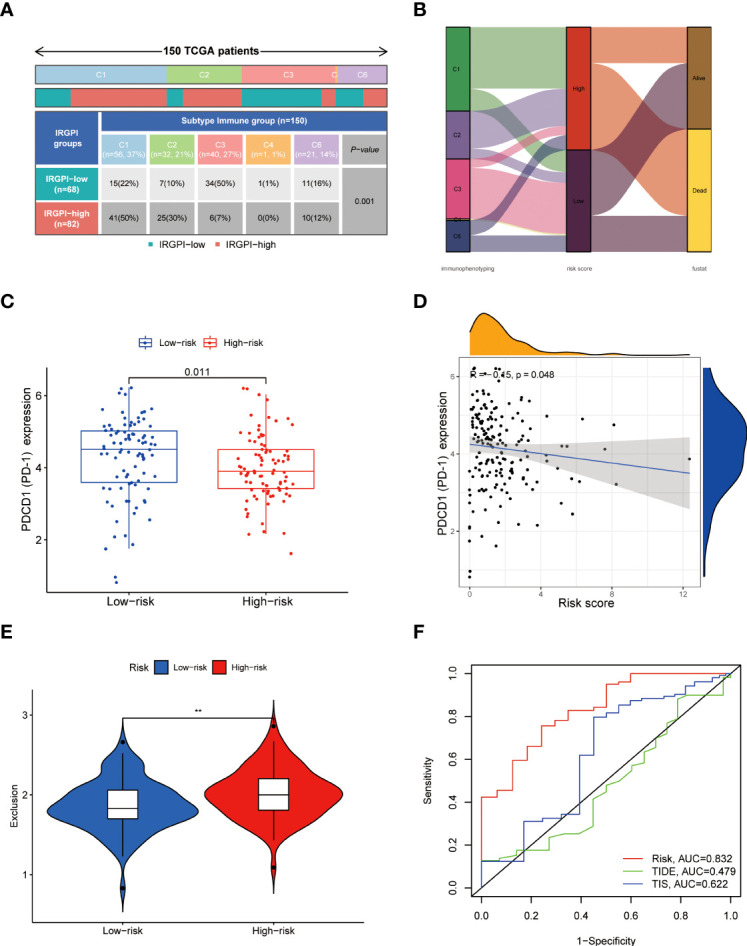
Relationship between HAPM and immunotherapy. **(A, B)** Distribution of immunophenotyping between different subgroups. **(C)** Differences in PD-1 expression between different subgroups (TCGA-PAAD). **(D)** Correlation between HAPM and PD-1 (TCGA-PAAD). **(E)** Differences in T-cell exclusion scores between different subgroups. **(F)** ROC analysis of HAPM, TIS and TIDE on 3-year OS of TCGA-PAAD dataset.

We found significant differences in the expression of HLA-B, HLA-G, CD160, HAVCR1, CD27, CD70, CD226 and CA9 in different subgroups (**Figures 8A–D**). Further analysis revealed differences in the distribution of immunophenotypes between different subgroups (**Figures 9A, B**). The expression of PD-1 was significantly lower in high-risk subgroup than in low-risk subgroup, and PD-1 expression correlated significantly with risk score (**Figures 9C, D**). High-risk subgroup had higher T-cell exclusion scores than those in the low-risk subgroup, suggesting that low-risk subgroup may be more suitable for immunotherapy (**Figure 9E**). ROC curves displayed predictive efficacy of HAPM was significantly better than that of the TIDE and TIS models (**Figure 9F**).

## Discussion

Most malignancies experience hypoxia, which is one of the key elements influencing the growth and spread of these diseases (23). Due to their rapid proliferation rate, PC cells are more susceptible to a hypoxic microenvironment, which encourages proliferation, metastasis, and the development of chemoresistance (24, 25). In tumor patients, hypoxia and hypoxia-induced angiogenesis are significant contributors to aggressiveness, treatment resistance, and poor prognosis (26). Angiogenesis, a critical stage in the creation of hematogenous metastasis and the evolution of tumors, requires intricate molecular regulatory mechanisms (27). A number of anti-angiogenic targeted medications have been employed in the clinical treatment of tumor patients, and inhibition of angiogenesis has now gained acceptance as a viable therapy strategy for cancers. Inhibiting angiogenesis is currently recognized as a workable treatment option for tumors after a number of anti-angiogenic targeted drugs were used in the clinical treatment of tumor patients.

To elucidate the regulatory mechanisms between hypoxia and angiogenesis in PC, we performed bioinformatic analysis of hypoxia and angiogenesis genes. In particular, IL-17 signaling pathway induces angiogenesis in tumors, both directly and indirectly, and inhibits apoptosis by activating westbound inflammatory factors (28). Although IL-17 enhances synoviocyte invasion in a hypoxic environment by upregulating MMP2 and MMP9, its function in tumor cells in a hypoxic environment is yet unknown (29). Vascular cell adhesion molecules are able to activate quiescent endothelial cells under pathological conditions, leading to angiogenesis (30). Hypoxia-inducible factors enhance the adhesion of cancer cells to vascular endothelial cells and thus promote tumor angiogenesis, progression and metastasis through various pathways (31). One of the most prevalent signaling pathways in patients with retinopathy during the angiogenic phase is ECM-receptor interactions, which are also believed to be linked to the growth of breast cancer (32, 33). TNF-mediated NF-B/VEGFA axis is able to contribute to tumor angiogenesis, and autocrine HIF-1 and TNF increase the invasive potential of prostate cancer cells PC3 in a hypoxic tumor microenvironment (34, 35).

In our research, we constructed HAPM consisting of seven hypoxia and angiogenesis genes (SERPINB7, KRT13, MMP3, KIF23, SEC31B, LY6D, LRP3). All SERPINB families are associated with tumor cell invasion, with SERPINB1, SERPINB5 and SERPINB7 being more strongly associated with invasion (36). By controlling the expression of c-myc, the oncogene KRT13 encourages tumor cell proliferation and invasion. MMP family is involved in normal and pathological biological processes in the body and plays the integral function in a variety of tumor cell functions including angiogenesis, epithelial mesenchymal transition and invasion (37). Jeremy et al. found that tumor-derived MMP3 was able to promote prostate cell proliferation and angiogenesis *in vitro (* 38). KIF family, a class of molecular motor proteins, is involved in a multitude of cellular biological processes and its abnormal expression is closely associated with tumorigenesis and progression (39). Gao et al. found that patients with pancreatic ductal adenocarcinoma who expressed high levels of KIF23 had a worse prognosis, and subsequent fundamental research identified that it promoted the proliferation of PC cells (40). SEC31B is crucial for the growth of the pollen wall, although its function in tumor cells is uncertain (41). In head and neck squamous cell carcinoma, LY6D is highly expressed, and it may speed up tumor growth by causing the release of angiogenic factors (42). High expression of LY6D was linked to the emergence of neck metastases and chemoresistance in laryngeal squamous cell carcinoma (43). A conserved class of transmembrane proteins called the LRP family controls a number of biological processes (44, 45). Recent research has linked it to the emergence of Alzheimer’s syndrome, but no studies have been discovered linking it to tumors (43).

We discovered substantial variations in the proportions of B cells naïve, CD8(+) T cells, activated NK cells, monocytes, Macrophages M0, and dendritic cells in the two subgroups. Hypoxia may be a major factor throughout the life cycle of B cells (46). Patients with advanced NSCLC likely to have worse prognoses if there are fewer B cells that are naive in the tumor microenvironment (47). The diminished function and depletion of CD8(+) tumor-infiltrating lymphocytes is a key barrier to immunotherapy in the treatment of cancer patients, and hypoxia is one of the main causes of diminished CD8(+) T-cell function (48). T-cell development is inhibited by VEGF, a crucial component of angiogenesis, and it is strongly linked to the reduction of CD8(+) T-cells (49). Allison et al. demonstrate that dipeptidyl peptidase increases the infiltration of CD8(+) T-cells and NK cells, which lowers growth rate of PC and increases the effectiveness of anti-PD1 treatment (50). Through the release of interleukin 35, PC cells recruit monocytes and macrophages to enhance tumor angiogenesis and proliferation (51). Macrophages are of three types, M0, M1 and M2, of which M0 can be polarized into M1 or M2. M1 macrophages are associated with a good prognosis, whereas M2 macrophages are associated with a poor prognosis (52). As antigen-presenting cells, dendritic cells are strongly related to the effectiveness of immunotherapy, and dendritic cell-based immunotherapy is currently being used in clinical trials in PC patients (53). Genetic alterations, especially the K-Ras mutation, carry the heaviest burden in the progression of pancreatic precursor lesions into pancreatic ductal adenocarcinoma (PDAC). The tumor microenvironment is one of the challenges that hinder the therapeutic approaches from functioning sufficiently and leads to the immune evasion of malignant pancreatic cells. Mastering the mechanisms of these two hallmarks of PDAC can help us deal with the obstacles in treatment (54).

We discovered that high-risk subgroup had significantly lower PD-1 expression than those in low-risk subgroup, and that T-cell depletion score was higher in high-risk group than in low-risk group by comparing the immune profiles of patients in the two subgroups. Therefore, we believe the model can be used to determine whether a patient is a candidate for immunotherapy as well as to forecast the prognosis of PC patients.

## Conclusion

In this study, we elucidated potential molecular mechanisms between hypoxia and angiogenesis that may be related to the IL-17 signaling pathway, cell adhesion molecules, ECM-receptor interactions, and TNF signaling pathway, obtained genes that can be used to treat PC, and constructed models that can be used to predict patient prognosis and benefit from immunotherapy. These results might be of assistance in basic research and clinical management of PC patients.

## Data availability statement

The raw data supporting the conclusions of this article will be made available by the authors, without undue reservation.

## Author contributions

HL developed the concept of the project. The data was collected and evaluated by XL, XY, and ZH with the help of all the other authors. All authors reviewed and discussed the results and contributed to the paper preparation. All authors have read and approved the manuscript.

## Supplementary material

Supplementary Figure 1 PC DEGs obtained using the GTEx and TCGA databases. **(A)** volcano map; **(B)** heat map

Supplementary Figure 2 KM survival curves of genes for which HPM was constructed and ROC curves for the models. **(A–F)** KM survival curves. **(G)** ROC curves of the hypoxia prognostic model.

Supplementary Figure 3 KM survival curves of genes for which APM was constructed and ROC curves of the models. **(A–E)** KM survival curves. **(F)** ROC curves of APMs.

Supplementary Figure 4 Construction of HAPM. **(A–E)** Prognostic models were constructed by univariate Cox, lasso, and multivariate Cox analyses. **(F)** ROC curves of HAPM.

Supplementary Figure 5 Differences in clinical characteristics in different subgroups. **(A, B)** Differences in T-stage and Stage in different subgroups.

Supplementary Table 1 Hypoxia-associated genes.

Supplementary Table 2 Angiogenesis-associated genes.

Supplementary Table 3 Results of the four subgroups.

Supplementary Table 4 Construction of HAPM.

Supplementary Table 5 Results of PPI network analysis.

Supplementary Table 6 Results of GO enrichment analysis.

Supplementary Table 7 Results of KEGG enrichment analysis.
