# Supplementary figures and images for: Identification of gene signatures related to hypoxia and angiogenesis in pancreatic cancer to aid immunotherapy and prognosis
Source: Front Oncol. 2023 Mar 30;13:1119763. doi: 10.3389/fonc.2023.1119763 (PMC10098147; doi:10.3389/fonc.2023.1119763)

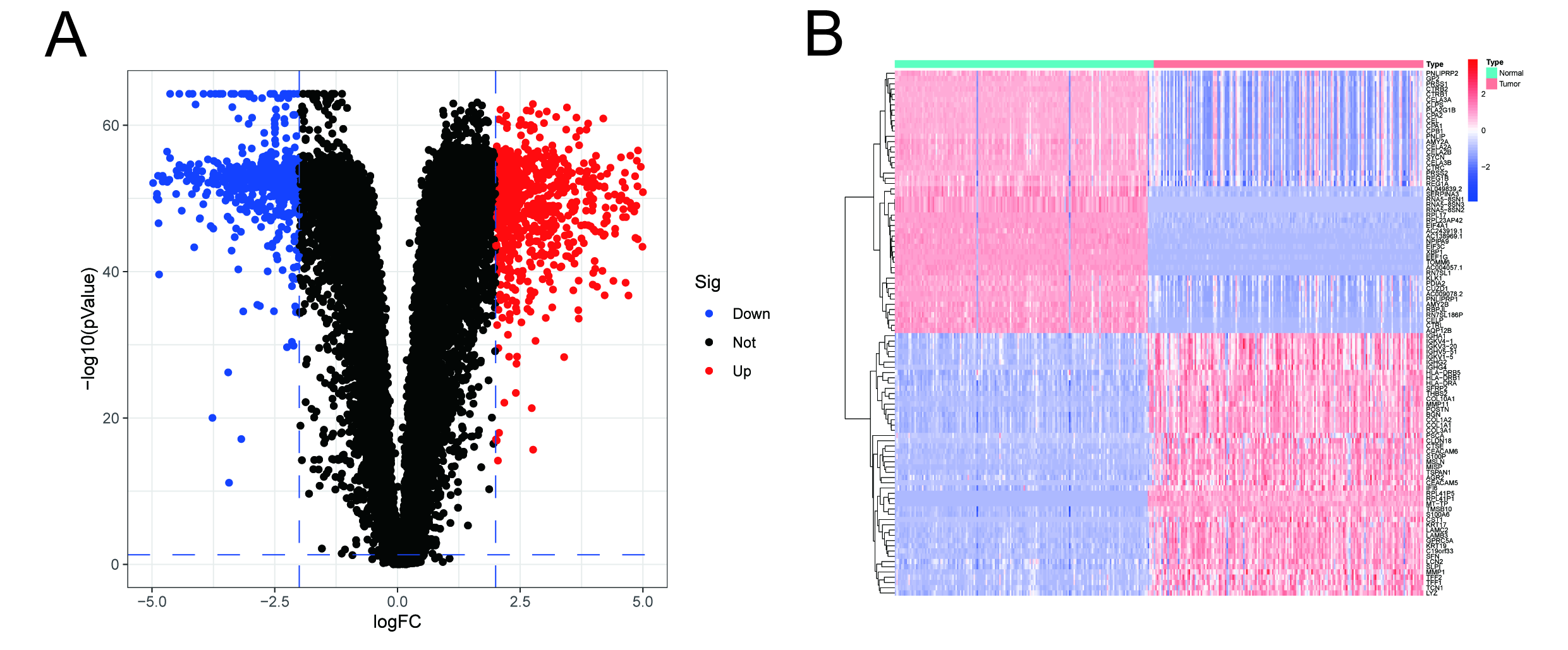

Supplement: Supplementary Figure 1 — PC DEGs obtained using the GTEx and TCGA databases. (A) volcano map; (B) heat map [file Image_1.tif]

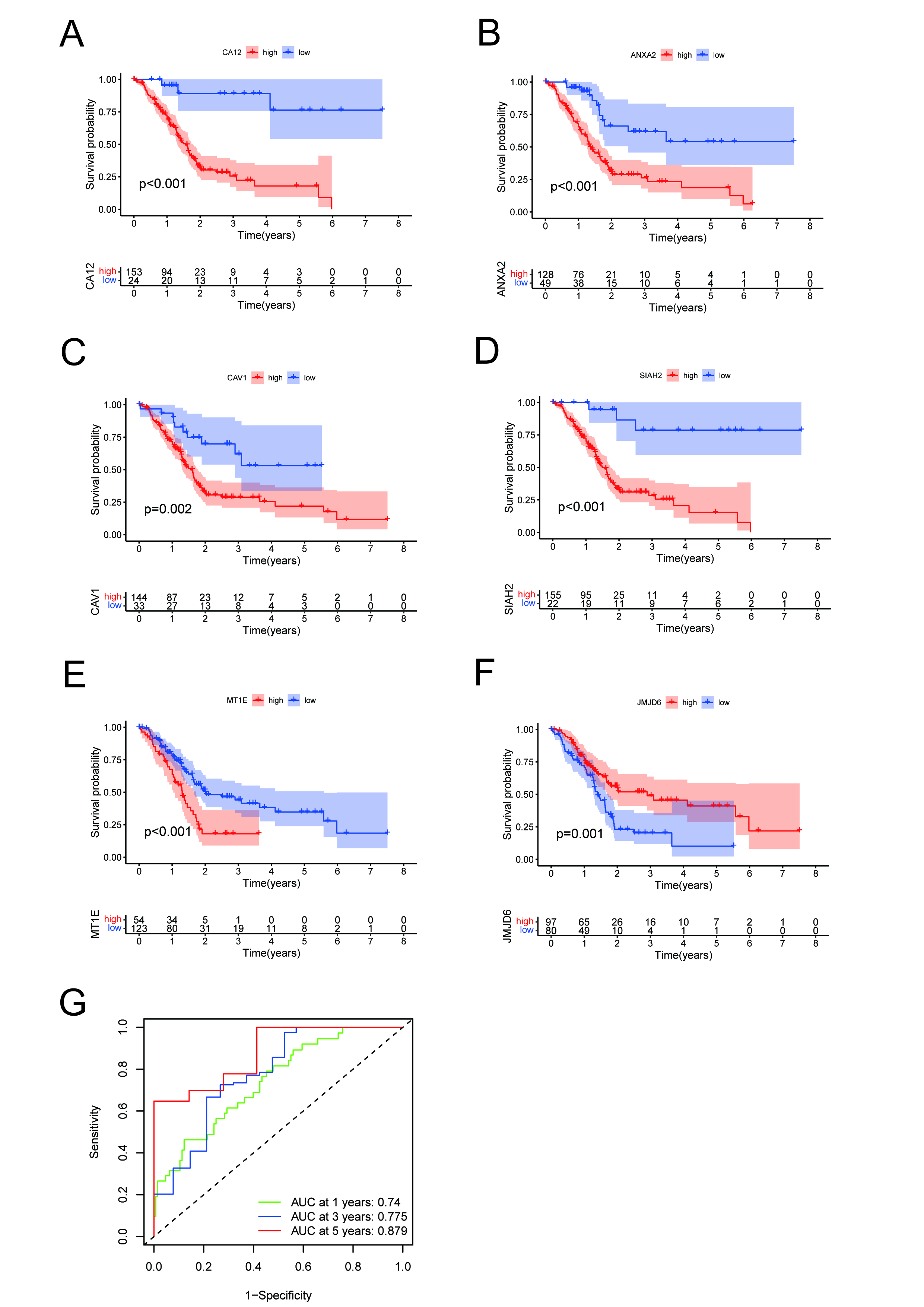

Supplement: Supplementary Figure 2 — KM survival curves of genes for which HPM was constructed and ROC curves for the models. (A–F) KM survival curves. (G) ROC curves of the hypoxia prognostic model. [file Image_2.tif]

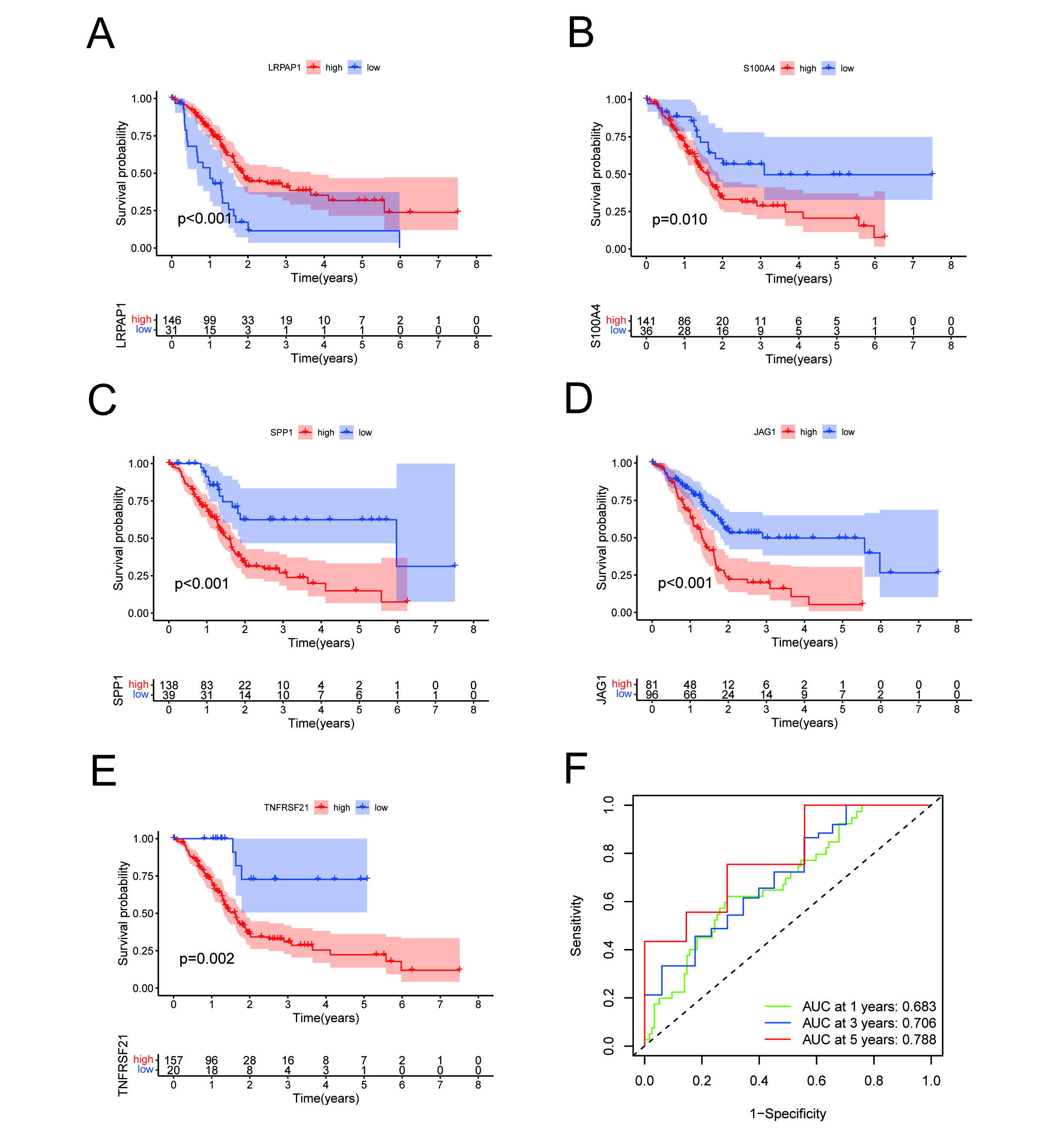

Supplement: Supplementary Figure 3 — KM survival curves of genes for which APM was constructed and ROC curves of the models. (A–E) KM survival curves. (F) ROC curves of APMs. [file Image_3.tif]

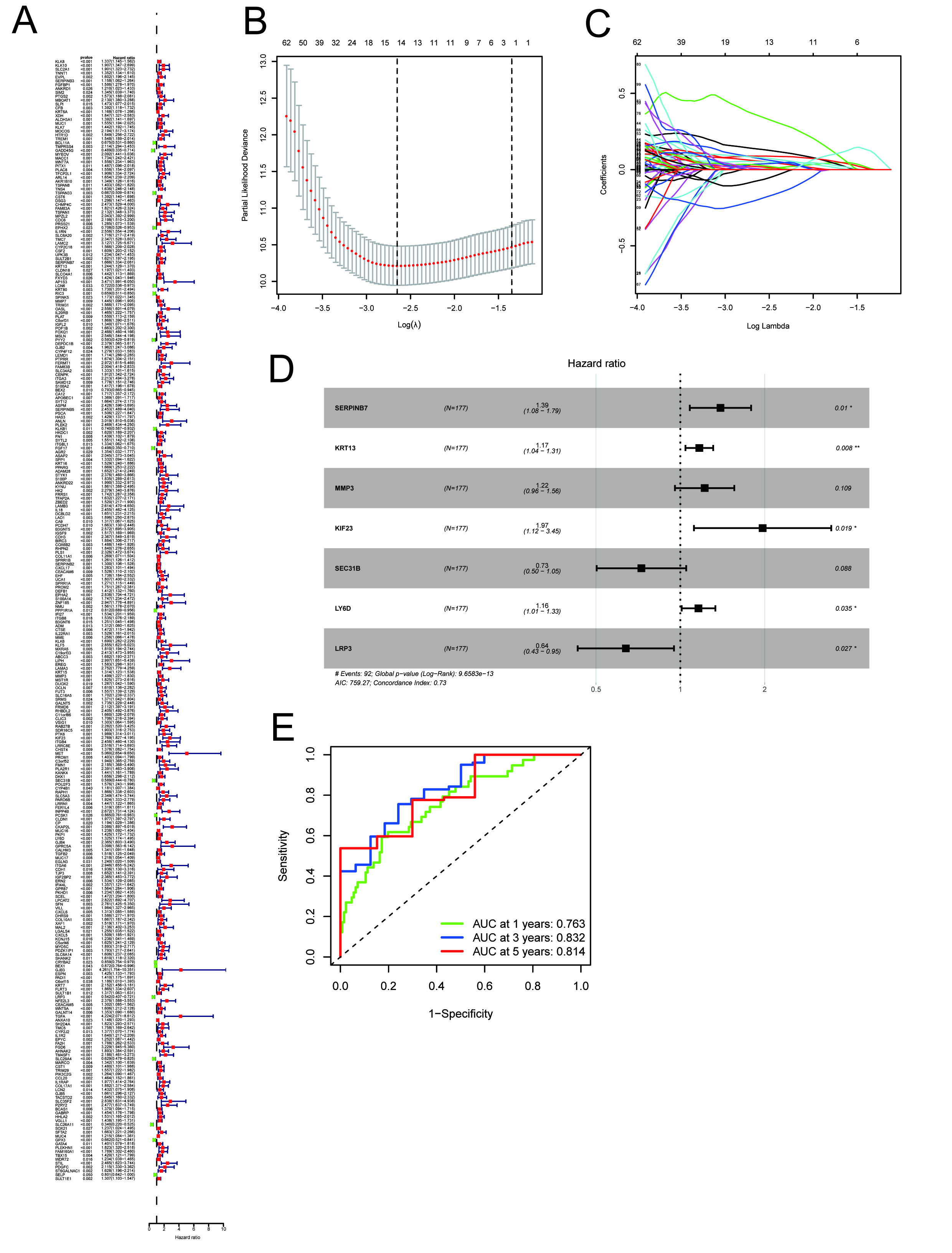

Supplement: Supplementary Figure 4 — Construction of HAPM. (A–E) Prognostic models were constructed by univariate Cox, lasso, and multivariate Cox analyses. (F) ROC curves of HAPM. [file Image_4.tif]

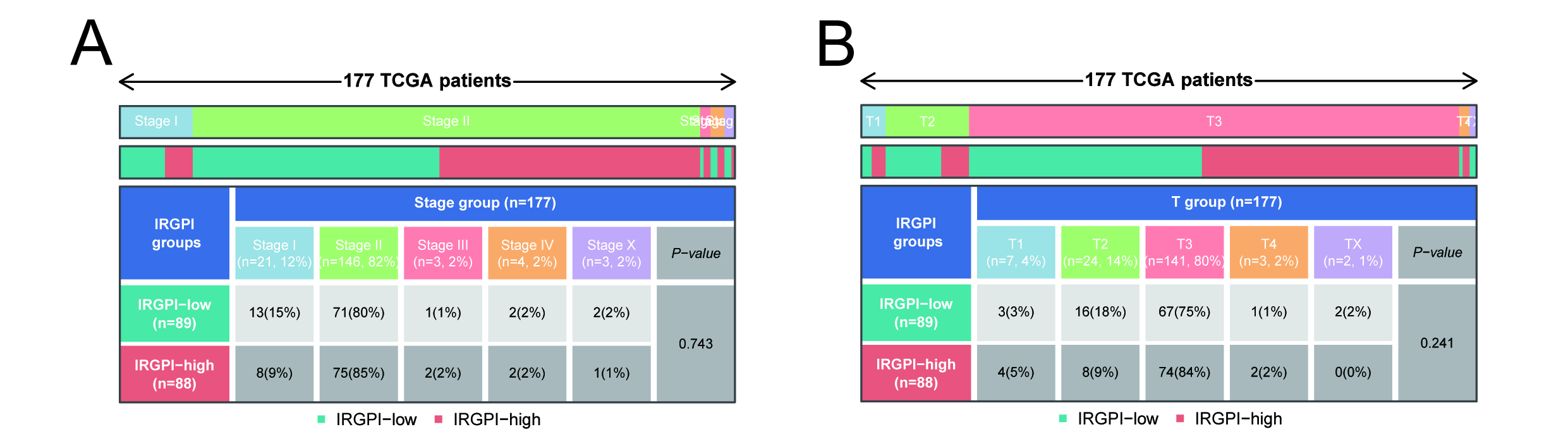

Supplement: Supplementary Figure 5 — Differences in clinical characteristics in different subgroups. (A, B) Differences in T-stage and Stage in different subgroups. [file Image_5.tif]
